# Acknowledgement to Reviewers of *Dentistry Journal* in 2014

**DOI:** 10.3390/dj3010014

**Published:** 2015-01-09

**Authors:** Dentistry Journal Editorial Office

**Affiliations:** MDPI AG, Klybeckstrasse 64, CH-4057 Basel, Switzerland

The editors of *Dentistry Journal* would like to express their sincere gratitude to the following reviewers for assessing manuscripts in 2014:

Addison, Owen
Ahmed, Junaid
Balkaya, Mehmet
Basrani, Bettina
Boffano, Paolo
Caneva, Marco
Crocombe, Leonard
Eick, Sigrun
Borzabadi-Farahani, Ali
Farahani, Shokoufeh Shahrabi
Fortin, Thomas
Frankenberger, Roland
Goiato, Marcelo C.
Goldstein, Moshe
Halazonetis, Demetrios J.
Hoang, Ha
Innes, Nicola
Kaye, Elizabeth Krall
Kielbassa, Andrej M.
Kim, Young-Kyun
Laskin, Daniel M.
Lin, Yng-Tzer
Majzoub, Zeina A.K.
Marshall, Teresa A.
Mazzitelli, Claudia
Mehndiratta, Mohit
Monje, Alberto
Norderyd, Ola
Perrotti, Vittoria
Peutzfeldt, Anne
Pillai, Ajay
Pommer, Bernhard
Rogers, John
Savabi, Omid
Scala, Alessandro
Schmidlin, Patrick
Schwass, Donald
Sigusch, Bernd W.
Silk, Hugh
Singh, Ayush Razdan
Sucasas, Luciane
Tauböck, Tobias
Thikkurissy, Sarat
Van den Engel-Hoek, Lenie
Wöstmann, Bernd
Yavuz, Izzet
Yuan, Kuo

